# Organic Waste from the Management of the Invasive *Oxalis pes-caprae* as a Source of Nutrients for Small Horticultural Crops

**DOI:** 10.3390/plants13172358

**Published:** 2024-08-23

**Authors:** Paula Lorenzo, Cristina Galhano, Maria Celeste Dias

**Affiliations:** 1 Associate Laboratory TERRA, Centre for Functional Ecology (CFE)—Science for People & the Planet, Department of Life Sciences, University of Coimbra, 3000-456 Coimbra, Portugal; celeste.dias@uc.pt; 2 Polytechnic Institute of Coimbra, Coimbra Agriculture School, Bencanta, 3045-601 Coimbra, Portugal; cicgalhano@esac.pt

**Keywords:** Bermuda buttercup, invasion control, organic fertilizer, photosynthesis, water deficit amelioration

## Abstract

The management of invasive plants is a challenge when using traditional control methods, which are ineffective for large areas, leading to the abandonment of invaded areas and the subsequent worsening of the situation. Finding potential uses for waste resulting from invaders’ management could motivate their control in the long-term, concurrently providing new bio-based resources with different applications. *Oxalis pes-caprae* is an invasive plant, widely distributed worldwide, which spreads aggressively through bulbils, creating a dense ground cover. This study was designed to assess the potential of *Oxalis* aboveground waste for use as fertilizer and in ameliorating deficit irrigation effects in growing crops. *Diplotaxis tenuifolia* (wild rocket) seedlings were planted in pots with soil mixed with *Oxalis* waste at 0, 2.2 and 4.3 kg m^−2^ or with commercial fertilizer, left to grow for 27 days and then irrigated at 100% or 50% field capacity for 14 days. The incorporation of the *Oxalis* waste improved the biomass, photosynthesis, sugars, total phenols and total antioxidant capacity in the crop, achieving commercial fertilization values, as well as increasing the phosphorus in soils. However, *Oxalis* waste seems not to directly affect plants’ relative water contents. Our results support the use of *Oxalis* waste as fertilizer, which can encourage the long-term control of this invasive species.

## 1. Introduction

Invasive plants are widely known to negatively impact ecosystem biodiversity, functioning and services in areas where they are not native [1,2,3,4]. The traditional management of invasive plants generally produces relatively good results in the short term but, in the long term, for large invaded areas, it often fails due to a lack of costly follow-up measures after the end of management projects [5]. Thus, invasive propagules that escape initial control efforts are able to recolonize cleared areas. To overcome this situation, several invasive-plant ecologists have recently suggested a more integrated and sustainable strategy to control the worst invasive plants, i.e., to find potential uses for the waste generated during control operations as a complementary way to reduce costs related to the management of these invasive plants [4,5,6].

*Oxalis pes-caprae* L. (Oxalidaceae) is a South African geophyte that has become invasive in several Mediterranean climatic regions of the world (e.g., [7,8,9]). This geophyte can grow up to 40 cm high and annually produces a basal rosette of leaves and yellow flowers organized in umbellate cymes [10]. In the European Mediterranean invader range, *O. pes-caprae* has a prolific level of vegetative reproduction, rapidly spreading through bulbils (e.g., [7,11,12,13]), and it is listed among the 10 worst invasive plants in terms of ecological and economic impacts [14]. It can expand over different types of habitat, such as disturbed, ruderal and agricultural areas, orchards, grasslands and abandoned fields [15]. The life-span of this species in Europe ranges from early autumn to late spring [16,17], resulting in a dense and homogeneous mat that outcompetes native plants. In fact, introduced *O. pes-caprae* tetraploids collected in the western Mediterranean basin can produce more aboveground biomass than *O. pes-caprae* tetraploids collected in their native range, in South Africa, when grown in competition with European species, suggesting an enhanced competitive ability [18]. In Spain, for example, *O. pes-caprae* can produce relatively high quantities of fresh aboveground biomass, at an average of 20.95 Mg ha^−1^ [13].

Although *O. pes-caprae* is considered a noxious weed, active management to control its invasion has been scarcely implemented in semi-natural ecosystems. Chemical formulations gave good results in controlling *O. pes-caprae* invasion in Australian agricultural fields (chlorsulfuron, [19]) and on a Mediterranean island (glyphosate [16]). However, the application of synthetic herbicides should be reduced due to toxic side effects [20]. Alternatively, the post-emergence application of the bioherbicide pelargonic acid showed good results in controlling *O. pes-caprae* in Mediterranean vineyards [21]. Other methods, such as the consumption of bulbs and tubers by pigs and turkeys, or the grazing of leaves by the noctuid moth, *Klugeana philoxalis*, have been suggested for Australian invaded areas [19,22]. However, no further information on the effectiveness of these methods has been reported. The control of invasions while feeding livestock could be useful for greatly reducing *O. pes-caprae* biomass, but this approach is discouraged, since the oxalate present in leaves produces poisoning if eaten in high quantities [23,24]. Recently, the management of olive orchards with a cover crop mixture (vetch/pea/barley) resulted in a reduction in *O. pes-caprae* density for three consecutive years [25]. Nevertheless, the authors of that study did not indicate whether the cover crop mixture also reduced the *O. pes-caprae* biomass.

On the other hand, the repeated mowing of biomass can contribute to bulbil exhaustion [26], without ecological, environmental or livestock health issues. The use of *O. pes-caprae* biomass resulting from control operations as a basis for creating new products may represent a complementary tool to make its management more attractive [5]. Several ecological studies revealed that *O. pes-caprae* has some fertilizing properties. For example, fresh biomass (leaves and flowers) of *O. pes-caprae* incorporated into soil, after a decomposition period, increased *Lactuca sativa* L. growth [13]. In addition, *O. pes-caprae* invasion can influence phosphorous (P) dynamics in soils, with invaded soils having higher contents of available P than non-invaded soils [27]. This is related to the release of oxalic acid from leaves after plant death and decomposition, as oxalate is a chelating agent that improves P availability is soils [27,28].

The main objective of this study was to assess whether the untreated above-ground biomass of *O. pes-caprae* in soil has potential value when it is applied as an organic fertilizer and/or whether it confers stress tolerance upon horticultural crops. To this end, we designed a greenhouse experiment in order to test the effects of different doses of *O. pes-caprae* biomass on the biometric and physiological biomarkers of a horticultural crop, *Diplotaxis tenuifolia* (wild rocket), under well or deficit irrigation conditions. We hypothesize that waste from *O. pes-caprae* biomass will improve the physiological performance of wild rocket by providing soil nutrients and some tolerance to water-deficit stress. The data provided by the present study can contribute to the creation of new products from *O. pes-caprae* waste, partially recovering funds allocated to its management, to reduce commercial fertilization inputs, to improve crops’ resilience to adverse environmental conditions, and to sequester carbon by incorporating waste biomass into soils.

## 2. Results

### 2.1. Plant Height, Biomass and Relative Water Content of Wild Rocket

**Figure 1:**
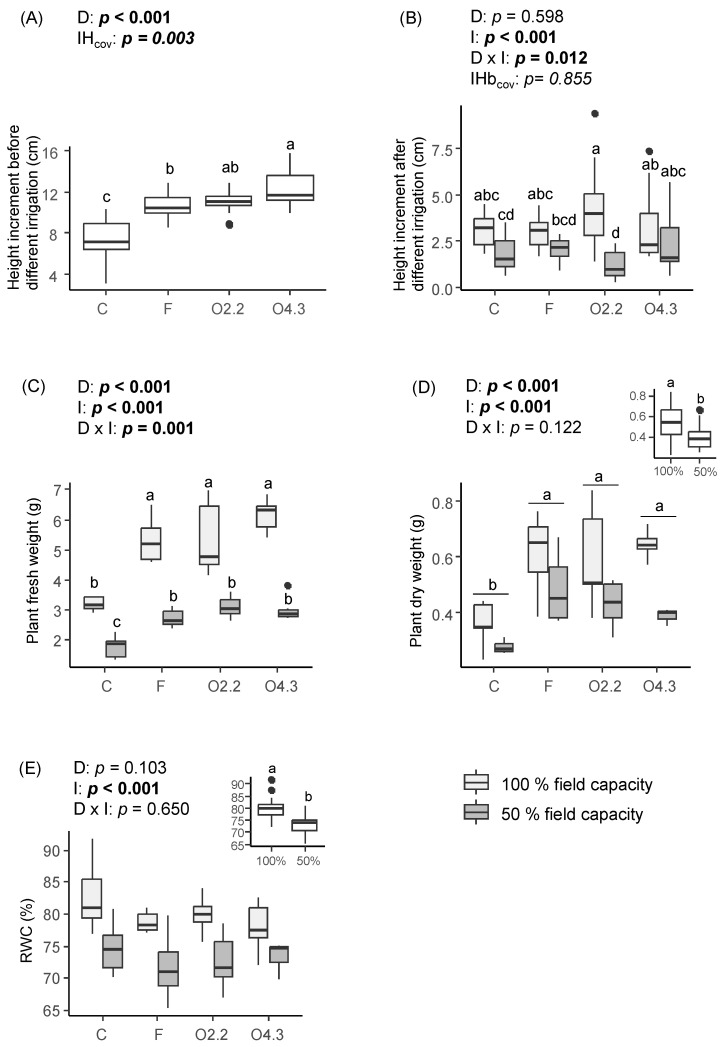
Effects of *Oxalis pes-caprae* waste dose (D), water irrigation (I) and the interaction between these two factors (D × I) on height increments before and after different irrigation (**A**,**B**), plant fresh and dry weight (**C**,**D**), and relative water content (RWC) (**E**) of the horticultural species *Diplotaxis tenuifolia*. Boxplots show the median values inside the boxes that bound the 25th and 75th percentile values. The dots represent values outside of the 10th and 90th percentile (represented by whiskers). IH_cov_: initial height covariable, IHb_cov_: initial height before different irrigation levels covariable, C: control, F: commercial fertilizer, O2.2: *Oxalis* waste at a dose of 2.2 kg m^−2^, O4.3: *Oxalis* waste at a dose of 4.3 kg m^−2^. Different letters indicate statistically significant differences at *p* ≤ 0.05 (in bold) level using general/generalized linear models followed by Sidak’s post hoc test, *n* = 7. When the interaction factor (D × I) is significant, statistical letters refer first to differences among C100%, C50%, F100%, F50%, O100%, and O50% treatments. Otherwise, statistical letters refer to differences between C, F, and O treatments when factor D is significant and/or to differences between 100% and 50% treatments (small figure inside the figure) when factor I is significant.

The height increment before different levels of water irrigation was significantly affected by *Oxalis* waste dose and the covariate (Figure 1A). The wild rocket plants growing with *Oxalis* waste and F were taller than the control plants (C). Additionally, *Oxalis* plants grown with waste at a dose of 4.3 kg m^−2^ (O4.3) were 1.6-fold taller than the plants grown with the commercial organic fertilizer (F) (Figure 1A). The height increment after the application of different water regimes was significantly affected by the water irrigation factor and the interaction between this factor and the *Oxalis* waste dose (Figure 1B). The O4.3 soil produced taller plants than the *Oxalis* waste at a dose of 2.2 kg m^−2^ (O2.2) under 50% field capacity irrigation (Figure 1B).

The *Oxalis* waste dose, water irrigation and *Oxalis* waste dose × water irrigation interaction had a significant effect on the fresh weights of wild rocket plants (Figure 1C). The plants growing in soils with *Oxalis* waste and commercial fertilization showed a higher increase in fresh weight (about 1.7-fold on average) than those in the control soils in both the 100% and the 50% water irrigation level (Figure 1C). However, the dry weight of wild rocket plants was only affected by the *Oxalis* waste dose and water irrigation (Figure 1D). This parameter was increased by 59–68% in O4.3, O2.2, and F compared with the control, and by 27% more at the 100% compared with the 50% irrigation level (Figure 1D).

Water irrigation significantly affected the relative water content (RWC) of the wild rocket plants, as it was higher in the well-watered plants (Figure 1E).

### 2.2. Gas Exchange, Chlorophyl a Fluorescence, and Pigment Contents of Wild Rocket

**Figure 2:**
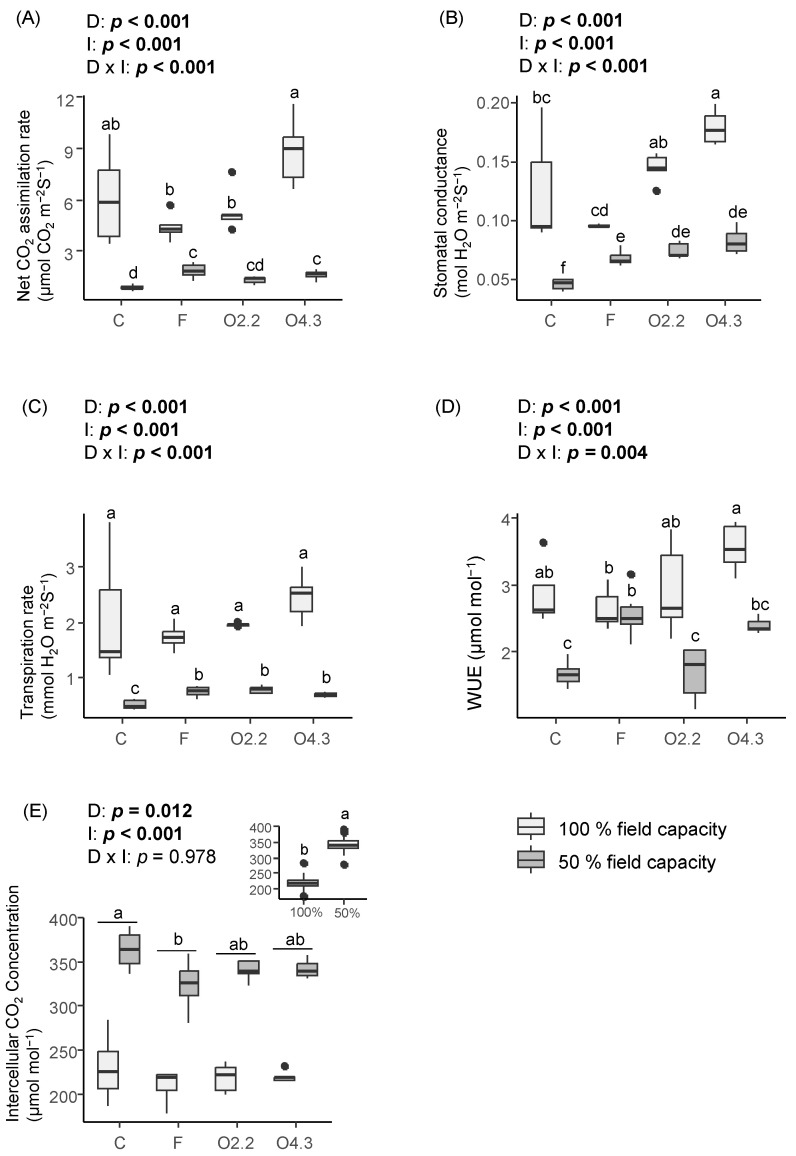
Effects of *Oxalis pes-caprae* waste dose (D), water irrigation (I), and the interaction between these two factors (D × I) on net CO_2_ assimilation rate (**A**), stomatal conductance (**B**), transpiration rate (**C**), water use efficiency (WUE) (**D**), and intercellular CO_2_ concentration (**E**) of the horticultural species *Diplotaxis tenuifolia*. Boxplots show the median values inside the boxes that bound the 25th and 75th percentile values. The dots represent values outside of the 10th and 90th percentile (represented by whiskers). C: control, F: commercial fertilizer, O2.2: *Oxalis* waste at a dose of 2.2 kg m^−2^, O4.3: *Oxalis* waste at a dose of 4.3 kg m^−2^. Different letters indicate statistically significant differences at *p* ≤ 0.05 (in bold) level using general/generalized linear models followed by Sidak’s post hoc test, *n* = 7. When the interaction factor (D × I) is significant, statistical letters refer first to differences among the C100%, C50%, F100%, F50%, O100%, and O50% treatments. Otherwise, statistical letters refer to differences between the C, F, and O treatments when factor D is significant and/or to differences between the 100% and 50% treatments (small figure inside the figure) when factor I is significant.

The *Oxalis* waste dose, water irrigation, and the interaction between these two factors significantly affected the net CO_2_ assimilation rate, stomatal conductance, transpiration rate, and water use efficiency (WUE) (Figure 2A, Figure 2B, Figure 2C and Figure 2D, respectively). However, the intercellular CO_2_ concentration was only affected by the *Oxalis* waste dose and water irrigation (Figure 2E). The net CO_2_ assimilation rate was 1.6- and 2-fold higher in the O4.3 treatment than in the O2.2 and F treatments, respectively, under 100% water irrigation, and 1.8- and 2.1-fold higher in the O4.3 and F treatments, respectively, than in the C under 50% field capacity (Figure 2A). Similarly, the wild rocket plants growing in the O4.3 soils showed increased stomatal conductance compared with those in the F and C soils under 100% irrigation (Figure 2B). This parameter was also increased by 1.5–1.89-fold in the plants from the F, O2.2, and O4.3 treatments compared to the control with 50% irrigation (Figure 2B). The F, O2.2, and O4.3 wild rocket plants also had 1.3–1.5-fold higher transpiration rate than the C plants under 50% irrigation (Figure 2C). Commercial fertilization improved the WUE of the plants compared to the O2.2 and C treatments under 50% field capacity (Figure 2D).

**Figure 3:**
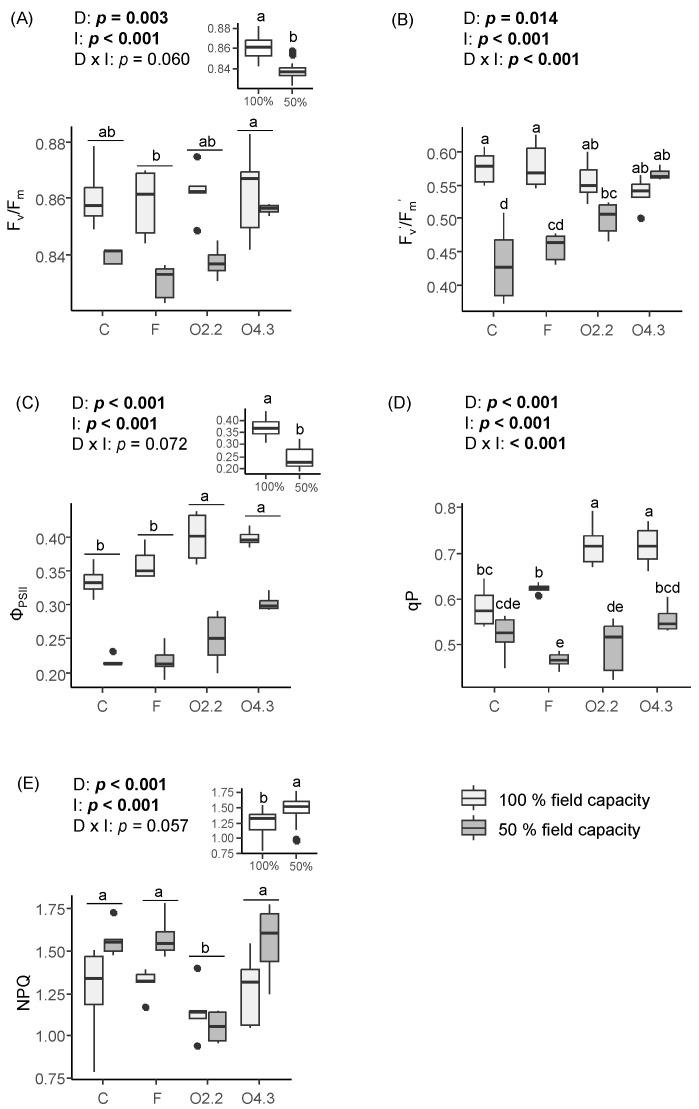
Effects of *Oxalis pes-caprae* waste dose (D), water irrigation (I), and the interaction between these two factors (D × I) on the maximum quantum efficiency of photosystem II (F_v_/F_m_) (**A**), efficiency of excitation energy capture by open PSII reaction centers (F_v_’/F_m_’) (**B**), effective efficiency of photosystem II (Φ_PSII_) (**C**), photochemical quenching (qP) (**D**), and non-photochemical quenching (NPQ) (**E**) of the horticultural species *Diplotaxis tenuifolia*. Boxplots show the median values inside the boxes that bound the 25th and 75th percentile values. The dots represent values outside of the 10th and 90th percentile (represented by whiskers). C: control, F: commercial fertilizer, O2.2: *Oxalis* waste at a dose of 2.2 kg m^−2^, O4.3: *Oxalis* waste at a dose of 4.3 kg m^−2^. Different letters indicate statistically significant differences at *p* ≤ 0.05 (in bold) level using general/generalized linear models followed by Sidak’s post hoc test, *n* = 7. When the interaction factor (D × I) is significant, statistical letters refer first to differences among the C100%, C50%, F100%, F50%, O100%, and O50% treatments. Otherwise, statistical letters refer to differences between the C, F, and O treatments when factor D is significant and/or to differences between the 100% and 50% treatments (small figure inside the figure) when factor I is significant.

The F_v_/F_m_, Φ_PSII_, and NPQ parameters were significantly affected by the *Oxalis* waste dose and water irrigation (Figure 3A, Figure 3C and Figure 3E, respectively). However, F_v_’/F_m_’ and qP were affected by the *Oxalis* waste dose, water irrigation, and the interaction between these two factors (Figure 3B and Figure 3D, respectively). The F_v_/F_m_ was highest in wild rocket plants growing in the O4.3 treatment and lowest in the plants growing in the F (Figure 3A). One hundred percent field capacity increased the F_v_/F_m_ (Figure 3A). By contrast, the wild rocket plants from the *Oxalis* waste treatments had higher F_v_’/F_m_’ than those from the C under 50% irrigation (Figure 3B). Additionally, the F_v_’/F_m_’ in the O4.3 plants was higher compared to that in the F plants (Figure 3B). The wild rocket plants growing in soils with *Oxalis* waste had Φ_PSII_ values that were 17–28% higher than those in the plants in the F and C soils (Figure 3C). The plants gown under 100% water irrigation also showed higher Φ_PSII_ values (Figure 3C). The *Oxalis* waste increased the qP by 15–23% in the wild rocket plants grown under 100% field capacity (Figure 3D). However, under 50% field capacity, only the O4.3 plants had higher qP compared to the F plants (Figure 3D). The application of *Oxalis* waste at 2.2 kg m^−2^ reduced the NPQ parameter and the 50% water regime increased this parameter (Figure 3E).

**Figure 4:**
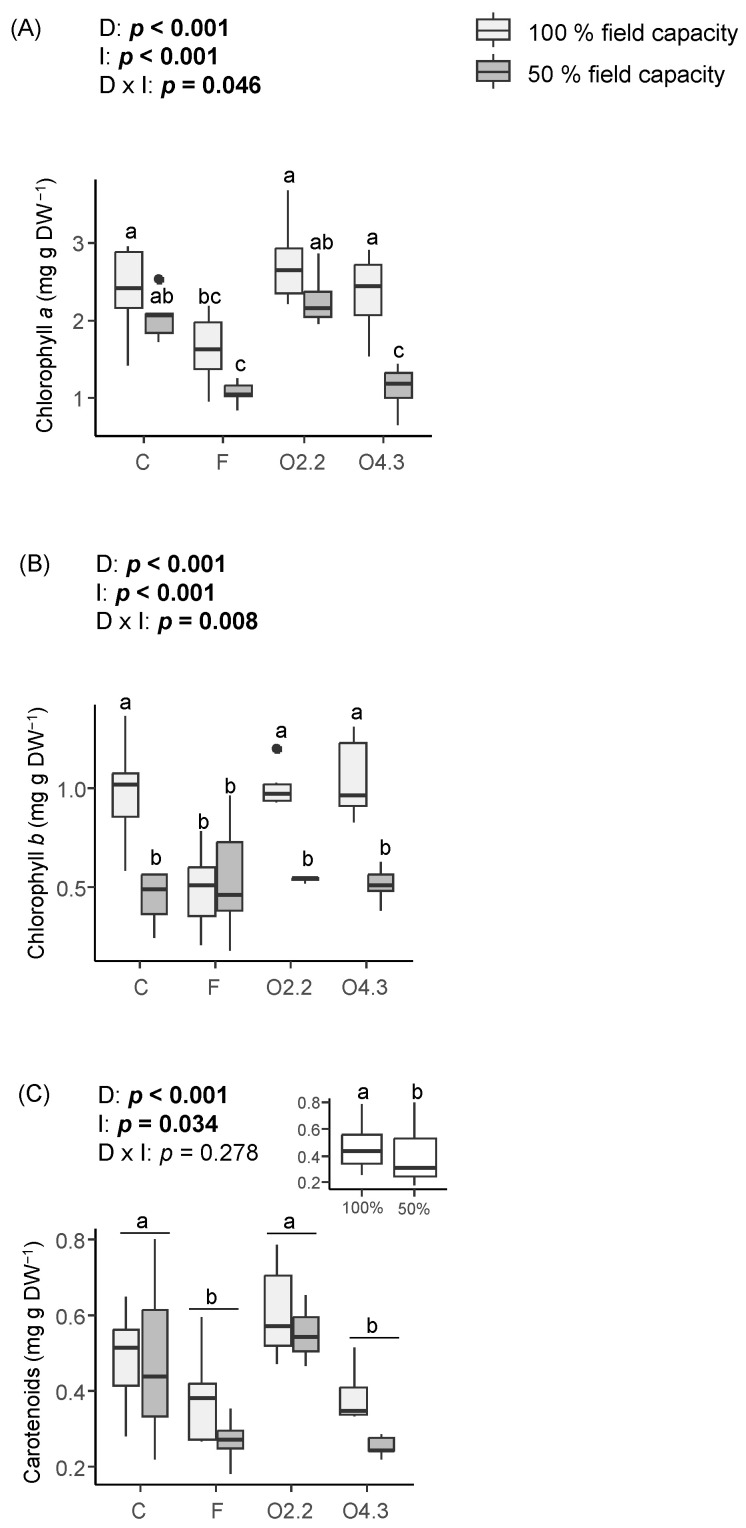
Effects of *Oxalis pes-caprae* waste dose (D), water irrigation (I), and the interaction between these two factors (D × I) on the contents of chlorophylls *a* and *b* (**A**,**B**) and carotenoids (**C**) in the horticultural species *Diplotaxis tenuifolia*. Boxplots show the median values inside the boxes that bound the 25th and 75th percentile values. The dots represent values outside of the 10th and 90th percentile (represented by whiskers). C: control, F: commercial fertilizer, O2.2: *Oxalis* waste at a dose of 2.2 kg m^−2^, O4.3: *Oxalis* waste at a dose of 4.3 kg m^−2^, DW: dry weight. Different letters indicate statistically significant differences at *p* ≤ 0.05 (in bold) level using general/generalized linear models followed by Sidak’s post hoc test, *n* = 7. When the interaction factor (D × I) is significant, statistical letters refer first to differences among the C100%, C50%, F100%, F50%, O100%, and O50% treatments. Otherwise, statistical letters refer to differences between the C, F, and O treatments when factor D is significant and/or to differences between the 100% and 50% treatments (small figure inside the figure) when factor I is significant.

The *Oxalis* waste dose, water irrigation, and the interaction between these two factors had a significant effect on the contents of chlorophyll *a* and *b* (Figure 4A and Figure 4B, respectively). The content of chlorophyll *a* was reduced in the wild rocket plants from the F treatment under 100% irrigation and in the plants from the F and O4.3 treatments under 50% irrigation (Figure 4A). Similarly, chlorophyll *b* was reduced in the plants growing in soils with F under 100% field capacity (Figure 4B). The content of carotenoids was affected by the *Oxalis* waste dose and water irrigation, as it was higher in the plants from the C and O2.2 treatments and in the well-watered plants (Figure 4C).

### 2.3. Total Soluble Sugars and Starch of Wild Rocket

**Figure 5:**
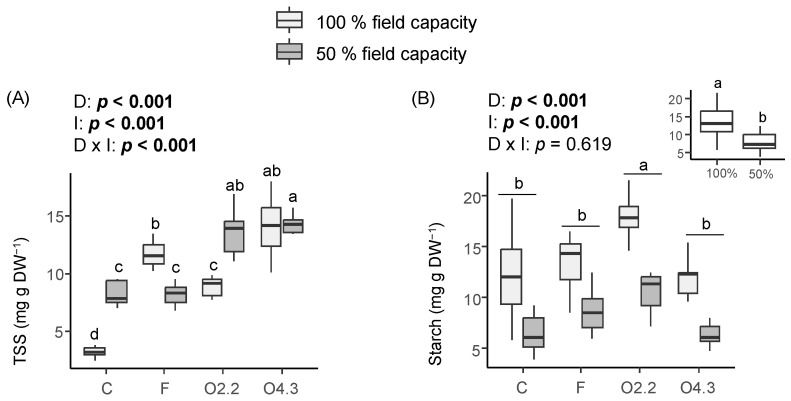
Effects of *Oxalis pes-caprae* waste dose (D), water irrigation (I), and the interaction between these two factors (D × I) on the contents of total soluble sugars (TSS) (**A**) and starch (**B**) in the horticultural species Diplotaxis tenuifolia. Boxplots show the median values inside the boxes that bound the 25th and 75th percentile values. C: control, F: commercial fertilizer, O2.2: *Oxalis* waste at a dose of 2.2 kg m^−2^, O4.3: *Oxalis* waste at a dose of 4.3 kg m^−2^, DW: dry weight. Different letters indicate statistically significant differences at *p* ≤ 0.05 (in bold) level using general/generalized linear models followed by Sidak’s post hoc test, *n* = 7. When the interaction factor (D × I) is significant, statistical letters refer first to differences among the C100%, C50%, F100%, F50%, O100%, and O50% treatments. Otherwise, statistical letters refer to differences between the C, F, and O treatments when factor D is significant and/or to differences between the 100% and 50% treatments (small figure inside the figure) when factor I is significant.

The content of total soluble sugars was significantly affected by the *Oxalis* waste dose, water irrigation, and *Oxalis* waste dose × water irrigation interaction (Figure 5A). The wild rocket plants growing in the O4.3, O2.2, and F soils accumulated 4.4, 2.8, and 3.7 times more total soluble sugars than those plants growing in the control soils under 100% irrigation conditions (Figure 5A). Similarly, the O4.3 and O2.2 plants produced 1.6–1.7 times more soluble sugars than the F and C plants under deficit water irrigation (Figure 5A). On the other hand, the content of starch was affected by the *Oxalis* waste dose and water irrigation factors, as it was higher in the plants growing in soils mixed with *Oxalis* waste at 2.2 kg m^−2^ and in the plants grown under 100% water irrigation (Figure 5B).

**Figure 6:**
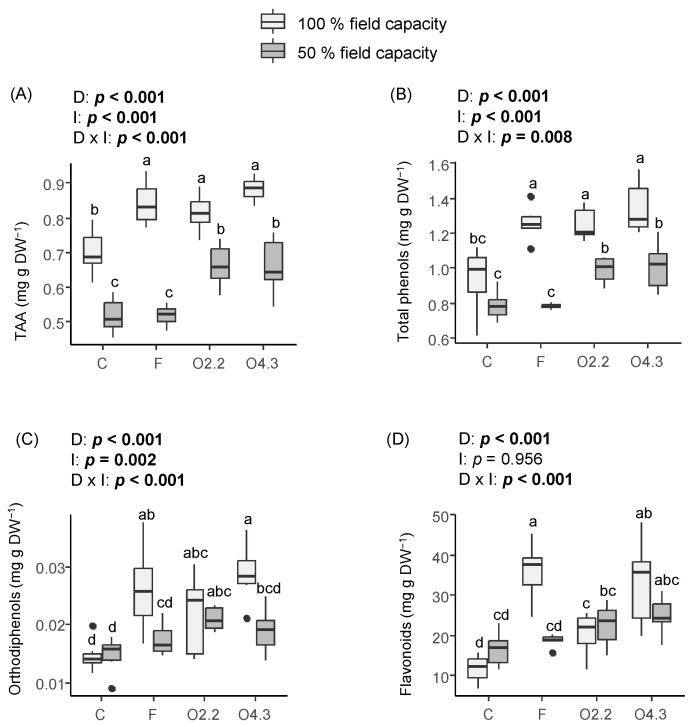
Effects of *Oxalis pes-caprae* waste dose (D), water irrigation (I), and the interaction between these two factors (D × I) on the total antioxidant activity (TAA) (**A**), total phenols (**B**), orthodiphenols (**C**), and flavonoids (**D**) in the horticultural species Diplotaxis tenuifolia. Boxplots show the median values inside the boxes that bound the 25th and 75th percentile values. The dots represent values outside of the 10th and 90th percentile (represented by whiskers). C: control, F: commercial fertilizer, O2.2: *Oxalis* waste at a dose of 2.2 kg m^−2^, O4.3: *Oxalis* waste at a dose of 4.3 kg m^−2^, DW: dry weight. Different letters indicate statistically significant differences at *p* ≤ 0.05 (in bold) level using general/generalized linear models followed by Sidak’s post hoc test, *n* = 7. When the interaction factor (D × I) is significant, statistical letters refer first to differences among the C100%, C50%, F100%, F50%, O100%, and O50% treatments.

The *Oxalis* waste dose, water irrigation, and the interaction between these two factors had a significant effect on the total antioxidant capacity (TAA), total phenols, and orthodiphenols (Figure 6A, Figure 6B and Figure 6C, respectively). However, the flavonoids content was only affected by the *Oxalis* waste dose and the interaction between this factor and water irrigation (Figure 6D). The wild rocket plants from the F and *Oxalis* waste treatments had a 16–43% higher TAA and total phenols contents than those from the control when a 100% irrigation level was applied (Figure 6A and Figure 6B, respectively). In addition, the TAA and total phenols of the wild rocket plants treated with the *Oxalis* waste was 26–28% higher than that in the F and C plants under reduced irrigation (Figure 6A and Figure 6B, respectively). The F and *Oxalis* waste treatments increased the of orthodiphenols contents in the well-watered plants (Figure 6C). However, this parameter was only increased by the O2.2 treatment under 50% irrigation (Figure 6C). By contrast, the wild rocket plants growing in soils with commercial fertilization and *Oxalis* waste at 4.3 kg m^−2^ had a 58–203% higher content of flavonoids compared to the C and O2.2 treatments when 100% water irrigation was applied, but no differences were found for this parameter under 50% irrigation (Figure 6D).

### 2.4. Soil Nutrients

**Figure 7:**
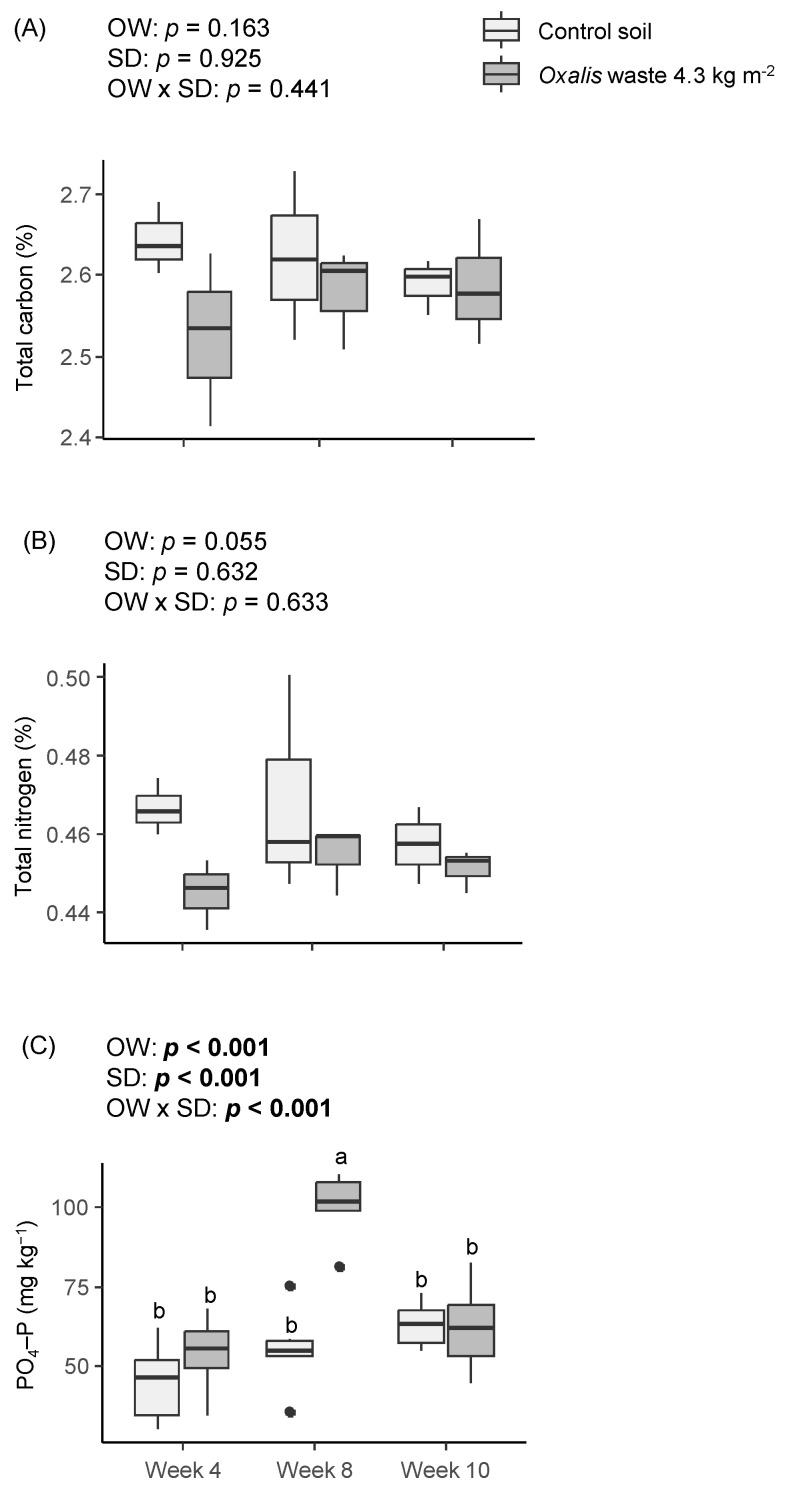
Effects of *Oxalis pes-caprae* waste (OW), the sampling date (SD), and the interaction between these two factors (OW × SD) on the total carbon (**A**), total nitrogen (**B**), and extractable phosphorous (**C**) in the soils during the *Oxalis* waste decomposition. Boxplots show the median values inside the boxes that bound the 25th and 75th percentile values. The dots represent values outside of the 10th and 90th percentile (represented by whiskers). Different letters indicate statistically significant differences at *p* ≤ 0.05 level (in bold) using repeated measured models followed by Sidak’s post hoc test, *n* = 6. When the interaction factor (OW × SD) is significant, statistical letters refer first to differences between the Week 4 control, Week 4 *Oxalis* waste, Week 8 control, Week 8 *Oxalis* waste, Week 10 control, and Week 10 *Oxalis* waste treatments. The absence of letters on a figure indicates that there are no significant statistical differences for any analysis factor.

The *Oxalis* waste, the sampling date, and the interaction between them did not affect the total carbon and nitrogen in the soil (Figure 7A,B). However, the content of extractable soil PO_4_–P was significantly affected by the *Oxalis* waste, the sampling date, and the *Oxalis* waste × sampling date interaction (Figure 7C). The soil mixed with the *Oxalis* waste and left to decompose for eight weeks almost doubled the amount of PO_4_–P compared to the other treatments (Figure 7C).

### 2.5. Multivariate Analysis

**Figure 8:**
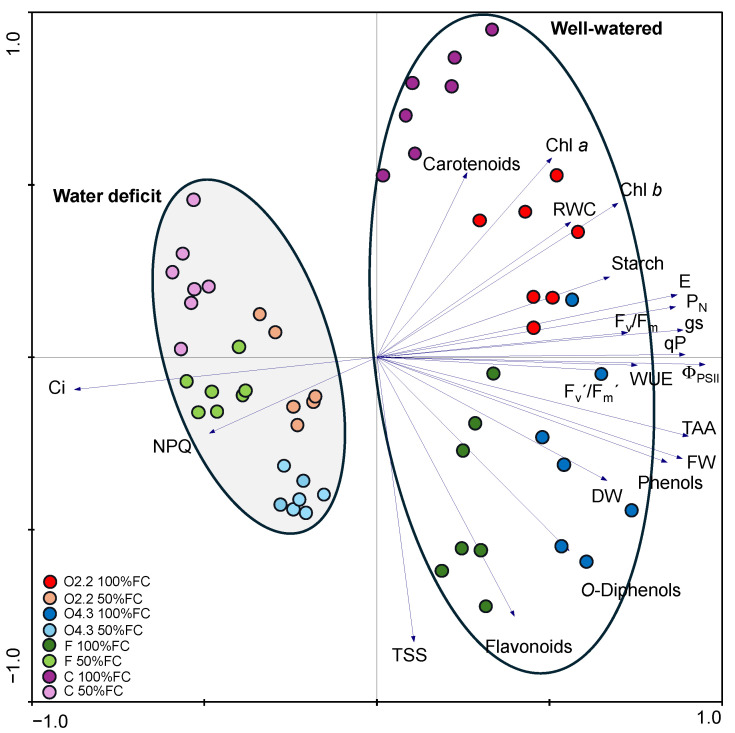
Principal component analysis (PCA) (x—first component and y—second component) of the physiological parameters in the wild rocket under well-watered and water deficit conditions. The first axis (PC1) explains 52% of the total variance and the second axis (PC2) explains 14% of the total variance. Colored circles represent sample scores and arrows show gradients resulting from water status, weight, pigments, carbohydrates, photosynthesis, oxidative stress, and antioxidant profiles. C: control, F: commercial fertilizer, O2.2: *Oxalis* waste at a dose of 2.2 kg m^−2^, O4.3: *Oxalis* waste at a dose of 4.3 kg m^−2^, FC: field capacity, Chl *a*: chlorophyll *a*, Chl *b*: chlorophyll *b*, Ci: intercellular CO_2_ concentration, DW: dry weight, E: transpiration rate, F_v_/F_m_: maximum quantum efficiency of photosystem II, F_v_’/F_m_’: efficiency of excitation energy capture by open PSII reaction centers, gs: stomatal conductance, FW: fresh weight, NPQ: non-photochemical quenching, P_N_: net CO_2_ assimilation rate, qP: photochemical quenching, RWC: relative water content, TAA: total antioxidant activity, TSS: total soluble sugars, WUE: water use efficiency, Φ_PSII_: effective efficiency of photosystem II.

The multivariate analysis (PCA) showed a clear separation between the well-watered (scores in the right quadrant) and water deficit (scores in the left quadrant) treatments, and among the *Oxalis* waste dose (C, O2.2, O4.3, F) treatments, suggesting a different physiological response of the wild rocket plants to the irrigation levels and *Oxalis* waste doses (Figure 8). Under well-watered conditions, the scores were more spread in the right quadrant, and the C scores were more separated from the F, O2.2, and O4.3. However, the water deficit treatment reduced the score spread, reducing the separation between the O2.2, O4.3, C, and F, and separated the O4.3 scores more from the F and O2.2 (which became closer). Under well-watered conditions, the O2.2 and O4.3 were better characterized by the high levels of Φ_PSII_ (Figure 8).

## 3. Discussion

In the present study, the application of *Oxalis* waste in soil affected the wild rocket’s physiology, modulating several biochemical and physiological processes, which led to a biomass production similar to that recorded in the treatment with commercial organic fertilizer. However, the *Oxalis* dose and the irrigation level influenced this response.

When water availability deviates from the needed quantity, it induces several relevant impairments, leading to weaker development and productivity losses in agriculture [29]. The leaf relative water content (RWC) is a physiological biomarker widely used to assess plants’ water status. In the present study, the reduction in irrigation of 50% induced a decrease in the stomata aperture (lower transpiration rate), but this protective mechanism was not effective in preventing leaf water loss, leading to a decline in the leaf RWC in the plants under all the treatments, indicating the impairment of the leaf water status. This reduction was accompanied by a general loss of plant fresh biomass, together with other physiological and biochemical alterations, suggesting a water deficit stress condition. However, although the F_v_/F_m_, an indicator of plant performance, was reduced by the 50% irrigation level, the values obtained were above 0.75, suggesting no significant reduction in the photosystem II efficiency [30]. This difference in the response profiles of the plants at 50% irrigation was notable in the multivariate analysis (Figure 8). The PCA analysis also highlighted that the O2.2 and O4.3 physiological responses were different from the C, and closer to the F, or even distinct, as in the case of the O4.3 under water deficit stress (Figure 8). The application of *Oxalis* waste to the wild rocket plants seems not to have influenced the RWC levels, but they affected other plant physiological and morphological traits according to the irrigation level and *Oxalis* waste dose. For instance, the beneficial application of O2.2 and O4.3 was noticeable in the photosynthesis, photosynthetic pigment contents, and carbohydrate levels. Under 100% irrigation, both *Oxalis* doses were effective in maintaining a net CO_2_ assimilation rate similar to or higher than those of the F and C plants, and the dose of O4.3 favored stomatal conductance more, despite the lack of changes registered in the intercellular CO_2_ availability of the mesophyll cells. Moreover, important differences in the light dependent reactions of photosynthesis were also observed. *Oxalis* waste seems to have enhanced the effective efficiency of photosystem II (possibly due to the maintenance of high levels of chlorophylls) and the photochemical quenching (Figure 8). Additionally, the O2.2 dose seems to have induced the accumulation of reserve carbohydrates (starch) and maintain the levels of protective pigments and carotenoids, similar to C plants, while the O4.3 dose acted similarly to the F plants, in terms of total soluble sugars accumulation and photoprotective processes related to NPQ.

In turn, under 50% irrigation, the *Oxalis* application enabled the plants to maintain a transpiration rate and a stomal conductance similar to those of the F plants, and higher than those of the C. Additionally, at the photochemistry level, the *Oxalis* waste acted mostly in the improvement of the efficiency of the excitation energy capture by the open PSII reaction centers and in photochemical quenching. These adjustments possibly enabled the plants to maintain a net CO_2_ assimilation rate (and WUE in O4.3 doses) similar to or higher than those of the F and C plants. Interestingly, both *Oxalis* doses promoted the accumulation of TSS, but only the O2.2 dose induced a higher investment in energy reserves (starch) and a reduction in the photoprotective mechanism related to NPQ.

Regarding the antioxidant status of the plants, under 100% irrigation, the O2.2 and O4.3 maintained TAA, total phenols, orthodiphenols, and flavonoids levels similar to those of the F plants, and higher than those of the C plants. However, under 50% irrigation, the *Oxalis* application in the soil favored the TAA and total phenols, indicating that these plants invested more in antioxidant protection.

All these physiological responses induced by the *Oxalis* waste application resulted in the production of biomass (plant fresh and dry mass) similar to that obtained when commercial fertilizer was used, and higher than the biomass produced in the C plants, under both irrigation levels. This effect was less obvious in terms of plant height investment (despite the differences noticed before the water deficit irrigation). This clearly indicates a beneficial effect of *Oxalis* waste application in soil, providing a growth and development effect similar to the commercial organic fertilizer. In line with our results, Lorenzo et al. [13] reported previously that the *Oxalis* waste present in soils promoted plant physiological performance (stem and root biomass accumulation). Our results also showed that the application of *Oxalis* waste increased the content of phosphorous (P) after eight weeks. This could be related to the fact that *Oxalis* biomass in soil decomposes rapidly, releasing high amounts of oxalic acid and increasing soil acidity [13,28]. In turn, oxalates in soils are associated with a high P available content, which could the improve P nutritional status of plants [28]. Phosphorus is the main element of several compounds, such as ATP, NADPH, sugar phosphates, phospholipids, and nucleic acids, and it is strictly related to photosynthesis, carbohydrate metabolism, and protein synthesis [31]. High P availability (P fertilizers) has been correlated with improved net CO_2_ assimilation rates, transpiration rates, and chlorophylls contents, and with the quantum efficiency of photosystem II [32,33,34,35]. In the present work, the increase in P availability in the O2.2 and O4.3 soils may explain the good performance of the wild rocket plants, inducing similar effects to the application of the commercial fertilizer.

The fact that the *Oxalis* waste biomass incorporation in the soil ultimately produced crops with similar characteristics to those obtained with the horticultural commercial fertilizer strongly suggests a use as organic fertilizer for the aboveground *Oxalis* waste. In invaded ranges, *O. pes-caprae* mainly reproduces asexually, through bulbils [11,12]. In addition, *O. pes-caprae* individuals from invaded ranges produce higher numbers of bulbils compared to individuals from the native South African range [18]. Each new bulbil will produce new offspring bulbils in the following season. The frequent movement of aboveground biomass during the growing season can lead to an exhaustion of bulbils [26] and reduce the number of new bulbils for the next generation (Dr. Mariana Castro, personal communication). Therefore, using *Oxalis* biomass as a source of nutrients for agricultural purposes can encourage active management to control *Oxalis* populations, which is urgently needed for persistent widely spread invasive plants [5]. However, despite not being the aim of the present study, this approach should be preferably completed with an economic analysis to evaluate the viability of the waste’s use in reducing *Oxalis* control costs [5,36].

## 4. Materials and Methods

### 4.1. Plant Material and Soil Collection and Preparation

Fresh aboveground waste (including leaves and flowers) from the invasive *Oxalis pes-caprae* management was collected at the Botanical Garden of the University of Coimbra, Portugal, in February 2023, cut into 2–5 cm long pieces and immediately used.

Seedlings of the horticultural species *Diplotaxis tenuifolia* (L.) DC (wild rocket) were obtained from a local supplier (Pombalverde Lda., Leiria, Portugal).

Agricultural soil (top soil, 20 cm layer) devoted to organic agriculture was collected at the Polytechnic Institute of Coimbra, Coimbra Agriculture School, Coimbra, Portugal, at the end of January 2023 and sieved through a 1 cm sieve to remove large debris and stones. Soil characteristics were described by Lorenzo et al. [37]. Soil was mixed with silica sand (0.7 mm diameter, AXTON SILICE) in a proportion of 3:1 *v*/*v* for the experiment.

### 4.2. Experimental Set-Up, Stress Implementation, and Biometric Parameters Analysis

We established a pot experiment with two factors: *Oxalis* waste at doses of 0 (negative control), 2.2, and 4.3 kg m^−2^ (C, O2.2, O4.3, respectively) [13] and irrigation level (100% and 50% soil water capacity) under greenhouse conditions with natural light at the University of Coimbra, Coimbra, Portugal. Commercial organic fertilizer (F, Adubo orgânico Siro BIO N-P-K 6-7-8, Portugal) at a recommended dose of 0.075 kg m^−2^ was used as a positive control. Each treatment had 14 replicates.

On 9 February 2023, 600 mL pots (10 cm in diameter) were filled with ~800 g of sand–soil mixed with *Oxalis* waste at abovementioned doses or only sand-soil. *Oxalis* waste was totally incorporated into the sand–soil mix. Next, pots were gently watered with tap water and left to decompose for four weeks to avoid phytotoxic effects [13]. Two seedlings of wild rocket were transplanted to each pot on 9 March 2023, thinned to one after six days and then allowed to grow for twenty-one days. Commercial fertilizer was added on sand–soil surface to F treatment at transplant. Pots were watered with tap water as needed. On 5 April 2023, all pots from all treatments were watered to 100% soil water capacity. Subsequently, well-watered pots continued to receive irrigation to 100% soil water capacity, while water deficit pots received only irrigation to 50% soil water capacity. Soil water content was assessed by weighting pots twice per week until the end of experiment, on 19 April 2023.

Maximum plant height was registered weekly from transplant to the end of the experiment and used to calculate the plant height increment as the difference between the last and the first records before and after different water irrigation regimes. At the end of experiment, half of the plants (*n* = 7) from each treatment were randomly selected and cut at aboveground level to determine fresh and dry (60 °C until constant weight) weights. The other half of plants per treatment were used to assess photosynthesis, leaf relative water content (RWC), pigment content, total soluble sugars, starch, total phenols, flavonoids, orthodiphenols, and antioxidant activity. Photosynthesis, RWC, and leaf nutrients were determined on fresh leaves. The remaining parameters were determined after collecting leaf samples, which were immediately frozen in liquid nitrogen and kept at −80 °C.

### 4.3. Determination of Wild Rocket Water Status, Photosynthesis, and Pigment Levels

Fresh leaves were weighted and, after incubation of the leaves in water for 48 h at 4 °C, the turgid weight was also determined. Leaf dry weight was determined after 10 days at 80 °C. Leaf relative water content was calculated as follows: (fresh weight − dry weight)/(turgid weight − dry weight) × 100.

Photosynthesis, gas exchange, and chlorophyll *a* fluorescence were determined in situ with a portable photosynthesis system LI-6400XT (LI-COR, Lincoln, NE, USA) coupled with a leaf chamber 6400-40 LCF with a light source (LED-based fluorescence and light source accessory for the LI-6400). Net CO_2_ assimilation rate (*Pn*), stomatal conductance (*g_s_*), transpiration rate (*E*), and the ratio of intercellular to extracellular CO_2_ concentration (C_i_/C_a_) were determined between 9 a.m. and 11 a.m., under 400 μmol mol^−1^ of CO_2_, light intensity of 300 µmol (photon) m^−2^ s^−1^, and temperature of ~24–25 °C. Water use efficiency (WUE) was calculated as WUE = *Pn*/*E*. Simultaneously, chlorophyll *a* fluorescence was also measured. Under light conditions, the steady-state fluorescence was averaged at 2.5 s, and the maximum fluorescence was established after the application of a saturating light pulse (0.8 s). Next, leaves were shaded and the F0′ was determined. Leaves were adapted to dark conditions for at least 40 min. A light saturating pulse (>5000 µmol photons m^−2^ s^−1^ for 0.8 s) was applied and the maximum quantum efficiency of photosystem II (F_v_/F_m_ = (F_m_ − F_0_)/F_m_) was determined. Additionally, the effective efficiency of photosystem II [Φ_PSII_ = (F_m_’ − F_s_)/F_m_’], efficiency of excitation energy capture by open PSII reaction centers [F_v_’/F_m_’ = (F_m_’ − F_0_)/F_m_’], photochemical quenching [qP = (F_m_’ − F_s_)/(F_m_’ − F_0_’)], and non-photochemical quenching [NPQ = (F_m_ − F_m_’)/F_m_’) were also calculated.

Chlorophyll *a* and *b* and carotenoid levels were determined according to Sims and Gamon [38]. Frozen leaves (approximately 50 mg) were ground with cold acetone/50 mM Tris pH 7.8 (80:20, *v*/*v*) and after centrifugation for 5 min at 5000× *g*, the absorbance of the supernatant was read at 470, 537, 647, and 663 nm with a spectrophotometer (Jenway^®^, model 7305, Cole-Parmer Ltd., Stone, UK). The pigment content was expressed as mg g dry weight (DW)^−1^.

### 4.4. Wild Rocket Carbohydrate Contents

Total soluble sugars were determined according to Irigoyen et al. [39]. Frozen leaf samples (approximately 80 mg) were ground with 5 mL of ethanol at 80% (*v*/*v*). After incubation for 1 h at 80 °C, an aliquot (10 µL) was homogenized with 250 µL of anthrone + water + H_2_SO_4_ and incubated at 100 °C for 10 min. After 10 min, the homogenates were cooled on ice and centrifuged for 10 min at 5000× *g*. The absorbance of the mixture was read at 625 nm in a HEALES MB-580 microplate reader (Shenzhen Huisong Technology Development Co., Ltd, Shenzhen, China). The content of total soluble sugars was determined using a calibration curve of glucose (y = 12.527x + 0.1174, r^2^ = 0.986) and expressed as mg g dry weight (DW)^−1^.

Starch content was determined according to Osaki et al. [40]. Frozen leaf samples (approximately 80 mg) were ground with 3 mL of perchloric acid (30%, *v*/*v*). After 1 h at 60 °C, an aliquot (10 µL) was homogenized with 250 µL of anthrone + water + H_2_SO_4_. After 10 min at 100 °C, the homogenate was cooled on ice and centrifuged for 10 min at 5000× *g*. The absorbance of the supernatant was read at 625 nm in a HEALES MB-580 microplate reader. The starch content was determined using a calibration curve of glucose (y = 15.237x + 0.1582, r^2^ = 0.989) and expressed as mg g dry weight (DW)^−1^.

### 4.5. Antioxidant Contents in Wild Rocket Leaves

Approximately 100 mg of frozen leaves were ground with 1.5 mL of methanol. After incubation for 30 min at 40 °C, the homogenate was centrifuged for 15 min at 5000× *g*. The supernatant (leaf methanolic extract) was collected and used for analysis of total antioxidant activity, total phenols, total flavonoids, and orthodiphenols. Total antioxidant capacity was determined using the ABTS^+•^ free cation radical scavenging activity method, according to Re et al. [41]. An aliquot of the leaf methanolic extract (10 µL) was homogenized with 400 µL of ABTS solution [2,20-azinobis(3-ethylbenzothiazoline-6-sulphonic acid)] and incubated for 10 min at 30 °C. The absorbance was read at 734 nm in a Thermo Scientific Multiskan FC spectrophotometer and the total antioxidant capacity was calculated using a calibration curve of gallic acid (y = 0.0011x + 0.00003, r^2^ = 0.994) and expressed as mg g dry weight (DW)^−1^. Total phenols were determined according to López-Orenes et al. [42]. An aliquot of the leaf methanolic extract (20 µL) was homogenized with 405 µL of Folin–Ciocalteu and 75 µL Na_2_CO_3_ (20%) and after incubation for 30 min at 37 °C, the absorbance of the mixture was measured at 765 nm in a Thermo Scientific Multiskan FC spectrophotometer (Waltham, MA, USA). Total phenols were calculated using a gallic acid calibration curve (y = 102.91x + 0.049, r^2^ = 0.984) and expressed as mg g dry weight (DW)^−1^. Total flavonoids were assessed based on López-Orenes et al. [42]. An aliquot of the leaf methanolic extract (37.5 µL) was homogenized with 37.5 µL of methanol. In total, 75 µL of NaNO_2_ (5%) was added and after mixing, 75 µL of AlCl_3_ (10%) was added. The mixture was agitated and after 6 min in dark, 125 µL of NaOH (1M) was added. The absorbance of the mixture was read at 510 nm in a Thermo Scientific Multiskan FC spectrophotometer. Total flavonoid content was calculated using a rutin calibration curve (y = 0.4186x + 0.1447, r^2^ = 0.998) and expressed as mg g dry weight (DW)^−1^. Orthodiphenols were determined according to the molybdate assay [43]. An aliquot of the leaf methanolic extract (80 µL) was mixed with 40 µL of sodium molybdate (5% *w*/*v* in 50% methanol) and 80 µL of methanol. After incubation for 15 min at 20 °C, the absorbance of the mixture was read at 370 nm in a Thermo Scientific Multiskan FC spectrophotometer. Orthodiphenol content was calculated using a gallic acid calibration curve (y = 3203.9x + 0.0396, r^2^ = 0.999) and expressed as mg g dry weight (DW)^−1^.

### 4.6. Evaluation of Soil Nutrients during Oxalis Waste Decomposition

Additionally, we conducted a pot experiment to determine the evolution of the main nutrients (nitrogen, N; carbon, C; and phosphorous, P) in soil during *Oxalis* waste decomposition without plant growth interference. On 9 February, 600 mL pots (10 cm in diameter) were filled with sand–soil mixed with *Oxalis* waste at a dose of 4.3 kg m^−2^ (O) or only sand–soil (C), with six replicates per treatment. Pots were left to decompose for 10 weeks (until 19 April 2023) and received full water as needed. Soil samples were collected at four, eight, and ten weeks after *Oxalis* waste incorporation, air-dried at room temperature, sieved through a 1 mm sieve and stored until soil analysis. Soil samples from the same treatment were pooled in pairs and an aliquot of each pair was used to estimate N, C, and P. Total soil N and C were estimated by dry combustion of ~100 mg soil with a CN 802 Elemental Analyzer (VELP, Scientifica, Usmate Velate, Italy; protein factor: none, O_2_ flow rate: 300 mL min^−1^, O_2_ factor: 0.7 mL mg^−1^). N and C results were obtained using standard aspartic acid and CaCO_3_ curves, respectively. We determined extractable soil PO_4_–P by extracting 2 g of soil with 40 mL of 2% acetic acid for 1 h of shaking, and then filtered it through a 0.45 μm filter, analyzed it for PO_4_–P following the molybdenum blue method [44], and read the absorbance of extracts at 700 nm. PO_4_–P results were obtained using a standard H_2_PO_4_ curve.

### 4.7. Statistical Analyses

Statistical analyses were conducted in R v4.3.3 [45]. In previous statistical analyses, we checked for normality of each dependent variable using the Shapiro–Wilk test (‘stats’ package). Next, we ran two-way general linear models (LMs, lm function, ‘stats’ package) for normal variables or generalized linear models (GLMs, glm function, ‘stats’ package) with Gaussian, Gamma or Inverse Gaussian error (link: log, identity or 1/mu^2^, respectively) in case of non-normal variables, to test for the effects of *Oxalis* waste dose (C, O2.2, O4.3, F), water irrigation (100 or 50% of field capacity), and the interaction between these two factors on fresh and dry aboveground biomass, photosynthetic parameters, RWC, pigments content, total soluble sugars, starch, total phenols, flavonoids, orthodiphenols, and antioxidant activity. We conducted a one-way GLM to explore the effect of *Oxalis* waste dose on height increment before different water regime with initial plant height as a covariable. Similarly, we ran a two-way GLM to evaluate the effect of *Oxalis* waste dose, water irrigation, and *Oxalis* waste dose × water irrigation interaction on height increment after different water regime with plant height before water irrigation treatment as a covariable. To test for the effect of *Oxalis* waste decomposition on the contents of total N and C and P over 10 weeks, we ran a two-way repeated measured model (lme function, ‘nlme’ package) with *Oxalis* waste (control and *Oxalis*), sampling date (week 4, week 8, and week 10), and the interaction between them as fixed factors and pot as random factor. The normality and homoscedasticity of residuals were checked after each model using the Shapiro–Wilk test and ‘DHARMa’ package. Post hoc mean comparisons were tested pairwise using the emmeans function (‘emmeans’ package) by comparing the emmeans obtained in each model. Additionally, a principal component analysis (PCA) was conducted using the program “CANOCO for Windows” v4.02 [46]. The level of significance was set at *p* ≤ 0.05.

## 5. Conclusions

In conclusion, the results of this work show that the application of *Oxalis* waste in soil improves wild rocket’s physiological performance according to the dose. *Oxalis* doses seem to act in several processes, such as photosynthesis, carbohydrate metabolism, and protective mechanism (antioxidants, NPQ, and carotenoids), modulating plant responses differently, but helping to respond similarly to the commercial organic fertilizer used. The obtained data support the use of this waste as a fertilizer under different irrigation levels, conferring some tolerance to water deficit stress. In turn, *Oxalis* application in soil seems not to influence plants’ relative water contents directly. The use of aboveground *Oxalis* biomass mowed during control operations as fertilizer can ultimately reboot the management of this broadly spread invasive species. Further studies are needed to perform a cost–benefit analysis of the process in order to reduce control costs.

## Data Availability

The data that support the findings of this study are available from the corresponding author, P.L., upon request.

## References

[1] Le Maitre D.C., Gaertner M., Marchante E., Ens E.J., Holmes P.M., Pauchard A., O’Farrell P.J., Rogers A.M., Blanchard R., Blignaut J. (2011). Impacts of invasive Australian acacias: Implications for management and restoration. Diver. Distrib..

[2] Pyšek P., Jarošik V., Hulme P.E., Pergl J., Hejda M., Schaffner U., Vila M. (2012). A global assessment of invasive plant impacts on resident species, communities and ecosystems: The interaction of impact measures, invading species’ traits and environment. Glob. Chang. Biol..

[3] Pyšek P., Hulme P.E., Simberloff D., Bacher S., Blackburn T.M., Carlton J.T., Dawson W., Essl F., Foxcroft L.C., Genovesi P. (2020). Scientists’ warning on invasive alien species. Biol. Rev..

[4] Souza-Alonso P., Rodriguez J., Gonzalez L., Lorenzo P. (2017). Here to stay. Recent advances and perspectives about *Acacia* invasion in Mediterranean areas. Ann. For. Sci..

[5] Lorenzo P., Morais M.C. (2023). Strategies for the management of aggressive invasive plant species. Plants.

[6] Ulm F., Estorninho M., de Jesus J.G., de Sousa Prado M.G., Cruz C., Máguas C. (2022). From a lose–lose to a win–win situation: User-friendly biomass models for *Acacia longifolia* to aid research, management and valorisation. Plants.

[7] Vilà M., Bartomeus I., Gimeno I., Traveset A., Moragues E.V.A. (2006). Demography of the invasive geophyte *Oxalis pes-caprae* across a Mediterranean island. Ann. Bot..

[8] Costa J., Ferrero V., Castro M., Jorge A., Afonso A., Loureiro J., Castro S. (2016). Pollen flow between flowers of the same morph in invasive populations of *Oxalis pes-caprae* L. in the western Mediterranean region. Plant Biosyst. Int. J. Deal. Asp. Plant Biol..

[9] Ferrero V., Navarro L., Castro S., Loureiro J., Sánchez J.M., Carvallo G.O., Barrett S.C. (2020). Global patterns of reproductive and cytotype diversity in an invasive clonal plant. Biol. Invasions.

[10] Pedraja O.S., Castroviejo S., Muñoz Garmendia F., Navarro C., Quintanar A., Buira A. (2015). *Oxalis* L.. Flora Iberica IX.

[11] Castro S., Ferrero V., Costa J., Sousa A.J., Castro M., Navarro L., Loureiro J. (2013). Reproductive strategy of the invasive *Oxalis pes-caprae*: Distribution patterns of floral morphs, ploidy levels and sexual reproduction. Biol. Invasions.

[12] Ferrero V., Barrett S.C.H., Castro S., Caldeirinha P., Navarro L., Loureiro J., Rodríguez-Echeverría S. (2015). Invasion genetics of the Bermuda buttercup (*Oxalis pes-caprae*): Complex intercontinental patterns of genetic diversity, polyploidy and heterostyly characterize both native and introduced populations. Mol. Ecol..

[13] Lorenzo P., González L., Ferrero V. (2021). Effect of plant origin and phenological stage on the allelopathic activity of the invasive species *Oxalis pes-caprae*. Am. J. Bot..

[14] Gallardo B. (2014). Europe’s top 10 invasive species: Relative importance of climatic, habitat and socio-economic factors. Ethol. Ecol. Evol..

[15] Gimeno I., Vila M., Hulme P.E. (2006). Are islands more susceptible to plant invasion than continents? A test using *Oxalis pes-caprae* L. in the western Mediterranean. J. Biogeogr..

[16] Lazzaro L., Ferretti G., Bianchi E., Benesperi R. (2019). Treatment by glyphosate-based herbicide allowed recovering native species after *Oxalis pes-caprae* L. invasion: Indications from a Mediterranean island. Plant Biosyst. Int. J. Deal. Asp. Plant Biol..

[17] BioDiversity4All. https://www.biodiversity4all.org/taxa/53169-Oxalis-pes-caprae.

[18] Tavares D., Loureiro J., Martins A., Castro M., Roiloa S., Castro S. (2019). Genetically based phenotypic differentiation between native and introduced tetraploids of *Oxalis pes-caprae*. Biol. Invasions.

[19] Pierce J.R. (1997). The biology of Australian weeds 31 *Oxalis pes-caprae* L.. Plant Prot. Q..

[20] Torretta V., Katsoyiannis I.A., Viotti P., Rada E.C. (2018). Critical review of the effects of glyphosate exposure to the environment and humans through the food supply chain. Sustainability.

[21] Muñoz M., Torres-Pagán N., Jouini A., Araniti F., Sánchez-Moreiras A.M., Verdeguer M. (2022). Control of problematic weeds in Mediterranean vineyards with the bioherbicide pelargonic acid. Agronomy.

[22] Kluge R.L., Claassens M. (1990). *Klugeana philoxalis* Geertsema (Noctuidae: Cuculliinae), the first potential biological control agent for the weed *Oxalis pes-caprae* L.. J. Entomol. Soc. South. Afr..

[23] Hulme P.E., Fernández-Palacios J.M., Morici C. (2024). Invasions, islands and impacts: A Mediterranean perspective. Island Ecology.

[24] Herbert E.W., Dittmer K.E. (2017). Acute and chronic oxalate toxicity in Miniature Horses associated with soursob (*Oxalis pes-caprae*) ingestion. Equine Vet. Educ..

[25] Volakakis N., Kabourakis E.M., Rempelos L., Kiritsakis A., Leifert C. (2022). Effect of different cover crops on suppression of the weed *Oxalis pes-caprae* L., soil nutrient availability, and the performance of table olive trees ‘Kalamon’ cv. in Crete, Greece. Agronomy.

[26] MITECO 2013 Catálogo Español de Especies Exóticas Invasoras. *Oxalis pes-caprae* L. OXAPES/EEI/FL06X. https://www.miteco.gob.es/content/dam/miteco/es/biodiversidad/temas/conservacion-de-especies/Oxalis_pes-caprae_2013_tcm30-69848.pdf.

[27] Sala A., Verdaguer D., Vilà M. (2007). Sensitivity of the invasive geophyte *Oxalis pes-caprae* to nutrient availability and competition. Ann. Bot..

[28] Cannon J.P., Allen E.B., Allen M.F., Dudley L.M., Jurinak J.J. (1995). The effects of oxalates produced by *Salsola tragus* on the phosphorous nutrition of *Stipa pulchra*. Oecologia.

[29] Xue J., San Zhou S., Wang W., Huo L., Zhang L., Fang X., Zhihong Yang Z. (2018). Water availability effects on plant growth, seed yield, seed quality in *Cassia obtusifolia* L., a medicinal plant. Agric. Water Manag..

[30] Dias M.C., Azevedo C., Costa M., Pinto G., Santos C. (2014). *Melia azedarach* plants show tolerance properties to water shortage treatment: An ecophysiological study. Plant Physiol. Biochem..

[31] Carstensen A., Herdean A., Schmidt S.B., Sharma A., Spetea C., Pribil M., Husted S. (2018). The Impacts of phosphorus deficiency on the photosynthetic electron transport chain. Plant Physiol..

[32] Tariq A., Pan K., Olatunji O.A., Graciano C., Li Z., Sun F., Sun X., Song D., Chen W., Zhang A. (2017). Phosphorous application improves drought tolerance of *Phoebe zhennan*. Front. Plant Sci..

[33] Attarzadeh M., Balouchi H., Rajaie M., Movahhedi Dehnavi M., Salehi A. (2019). Growth and nutrient content of *Echinacea purpurea* as affected by the combination of phosphorus with arbuscular mycorrhizal fungus and pseudomonas florescent bacterium under different irrigation regimes. J. Environ. Manag..

[34] Khan F., Siddique A.B., Shabala S., Zhou M., Zhao C. (2023). Phosphorus plays key roles in regulating plants’ physiological responses to abiotic stresses. Plants.

[35] Kayoumu M., Iqbal A., Muhammad N., Li X., Li L., Wang X., Gui H., Qi Q., Ruan S., Guo R. (2023). Phosphorus availability affects the photosynthesis and antioxidant system of contrasting low-P-tolerant cotton genotypes. Antioxidants.

[36] Ortega Z., Romero F., Paz R., Suárez L., Benítez A.N., Marrero M.D. (2021). Valorization of invasive plants from Macaronesia as filler materials in the production of natural fiber composites by rotational molding. Polymers.

[37] Lorenzo P., Guilherme R., Barbosa S., Ferreira A.J., Galhano C. (2022). Agri-food waste as a method for weed control and soil amendment in crops. Agronomy.

[38] Sims D., Gamon J. (2002). Relationships between leaf pigment content and spectral reflectance across a wide range of species, leaf structures and developmental stages. Remote Sens. Environ..

[39] Irigoyen J.J., Emerich D.W., Sanchezdiaz M. (1992). Water stress induced changes in concentrations of proline and total soluble sugars in nodulated alfalfa (*Medicago sativa*) plants. Physiol. Plant..

[40] Osaki M., Shinano T., Tadano T. (1991). Redistribution of carbon and nitrogen compounds from the shoot to the harvesting organs during maturation in field crops. Soil Sci. Plant Nutr..

[41] Re R., Pellegrini N., Proteggente A., Pannala A., Yang M., Rice-Evans C. (1999). Antioxidant activity applying an improved ABTS radical cation decolorization assay. Free Radic. Biol. Med..

[42] López-Orenes A., Dias M.C., Ferrer M.A., Calderón A., Moutinho-Pereira J., Correia C., Santos C. (2018). Different mechanisms of the metalliferous *Zygophyllum fabago* shoots and roots to cope with Pb toxicity. Environ. Sci. Pollut. Res..

[43] Giertych M.J., Karolewski P., de Temmerman L.O. (1999). Foliage age and pollution alter content of phenolic compounds and chemical elements in *Pinus nigra needles*. Water Air Soil Pol..

[44] Allen S.E., Grimshaw H.M., Rowland A.P., Moore P.D., Chapman S.B. (1986). Chemical Analysis. Methods of Plant Ecology.

[45] R Development Core Team (2015). R: A Language and Environment for Statistical Computing.

[46] ter Braak C.J.F., Smilauer P. (1998). CANOCO Reference Manual and User’s Guide to Canoco for Windows—Software for Canonical Community Ordination (Version 4).

